# Effect of up to 30-days of storage at different temperatures on detection of feline kidney injury molecule-1 in urine

**DOI:** 10.1186/s12917-022-03489-w

**Published:** 2022-11-04

**Authors:** Aleksandra Milaszewska, Alice Defarges, Michelle Oblak, Brigitte Brisson, Gabrielle Monteith, Dorothee Bienzle

**Affiliations:** 1 Department of Clinical Studies, University of Guelph, Guelph, ON N1G 2W1 Canada; 2 Department of Pathobiology, University of Guelph, Guelph, ON N1G 2W1 Canada

**Keywords:** Acute kidney injury, Biomarker, Chronic kidney disease, KIM-1, LFA, Renal failure, Validation

## Abstract

**Background:**

In humans, kidney injury molecule-1 (KIM-1) is a biomarker of acute kidney injury that can be quantified in urine. Preliminary investigation in cats with experimentally induced acute kidney injury showed that KIM-1 urine concentration correlated with kidney injury histopathology scores. A lateral flow assay (LFA) has recently become available for patient-side feline KIM-1 measurement. In vitro parameters of the assay have not yet been determined. The objectives of this study were to determine detection of KIM-1 in urine stored at different temperatures over time, to establish the linear range of the LFA, and to assess the intra-assay repeatability of measurements.

**Results:**

Ten urine samples with a range of KIM-1 concentrations were stored at room temperature (22^o^ C), 4^o^ C or -20^o^ C, and tested with the LFA on days 0, 1, 2, 3, 7, 14, and 30. The concentration of KIM-1 in samples was not significantly different from the day 0 value, except one sample that had been stored for 30 days at room temperature yielded a significantly higher value. The assay results had a correlation coefficient of 0.922. The mean coefficient of variation for all samples was 15.7%. The slope of the curve of expected versus measured values in samples diluted two-fold nine times was 0.908, and results were linear over all dilutions.

**Conclusions:**

The LFA for feline KIM-1 yields consistent results from stored urine samples. These characteristics will allow for KIM-1 to be measured retrospectively if immediate testing is not feasible. Within assay precision was high, and linearity over 9 logs of dilution suggests suitability for a range of subclinical and clinical kidney injuries.

**Supplementary Information:**

The online version contains supplementary material available at 10.1186/s12917-022-03489-w.

## Background

Chronic kidney disease (CKD) is a common condition of cats that is debilitating and progressive. It has been estimated that between 2 and 80% of cats ≥ 12 years old will develop CKD [1–3]. The causes of such high prevalence in some studies are not fully understood but it has been hypothesized that infectious agents, metabolic factors, renal ischemia, and nephrotoxic substances contribute to multiple subclinical injuries, and that ensuing inflammation and repair eventually result in interstitial fibrosis and clinically overt kidney disease [4, 5]. Acute kidney injury (AKI) most often manifests as a sudden decrease in glomerular filtration rate (GFR), which may be subclinical [6]. There is increasing awareness that even such subclinical AKI may lead to CKD, and that cats with CKD are at increased risk of additional injury (acute-on-chronic kidney disease, ACKD) [5, 7]. Considering the high prevalence of CKD, AKI in cats is probably often undiagnosed.

Historically, increasing serum creatinine concentration (SCC) was the mainstay for diagnosing AKI. Over time, limitations of SCC such as dependence on muscle mass and marked inter-individual variability became apparent. Thence, in humans, urine production was also considered in the staging of AKI [8]. However, SCC has a non-linear relationship with GFR, and decreased urine production is not a consistent indicator of the severity of AKI. Over the past decade, biomarkers of AKI such as kidney injury molecule-1 (KIM-1), neutrophil gelatinase-associated lipocalin (NGAL), cystatin C, clusterin, IL-18, and many others, have been evaluated in humans, and a few of these were described in companion animals [9–13]. Of these, KIM-1, is a transmembrane glycoprotein that was discovered as the first non-myeloid cell scavenger receptor [14]. Post AKI, surviving renal tubular epithelial cells expressing KIM-1 phagocytose necrotic debris in the tubular lumen by binding to apoptotic cells and triggering internalization [14]. The ectodomain of KIM-1 is cleaved off during this process and is shed into urine [15, 16]. In a rat ischemia–reperfusion (IR) model, ten minutes of bilateral ischemia induced by clamping the renal artery resulted in a fivefold increase of urine KIM-1 at 24 h without a concurrent increase in SCC or development of proteinuria [17]. When different biomarkers were compared, in humans KIM-1 outperformed other biomarkers in multiple clinical scenarios [18, 19]. From a meta-analysis it was estimated that the sensitivity and specificity of urine KIM-1 for the diagnosis of AKI was 74.0 and 86.0%, respectively, with 95% confidence intervals (CI) of 61-84% and 74-93% [20].

In feline kidneys, KIM-1 localized predominantly to the S3 segment of the proximal tubule [12, 21]. While the KIM-1 amino acid sequence is similar between humans, rats and cats, existing lateral flow assays (LFA) were insufficiently sensitive and specific to measure feline KIM-1 [22]. Therefore, monoclonal antibodies to recombinant feline KIM-1 were generated and a feline-specific LFA for KIM-1 was designed [22]. Results of the LFA are quantified with an optical reader equipped with a radio-frequency identification card (RFID).^1^ An internal positive control is measured during each assay, and results are read after 15 min of incubation. The final result is expressed as a ratio of the control and test sample [22].

The in vitro parameters of the feline KIM-1 LFA have not been established. In vitro test validation is an essential step for a diagnostic assay and may include assessment of linearity, precision, accuracy, analytical range, detection limits and interferences [23]. The features of a test that should be validated are largely dictated by the intended use of the test or method [23]. The KIM-1 LFA is not yet in clinical use, and this study addresses some aspects of validation. Therefore, the first aim of this study was to assess the effect of storage at different temperatures on the detectability of KIM-1 in urine. The second aim was to determine the intra-assay repeatability of KIM-1 measurements, and the third aim was to determine the linear range of the test. We hypothesized that KIM-1 is stable for at least 48 h in refrigerated (4 oC) or frozen (-20 oC) urine, but that it would be unstable if stored at room temperature for more than 24 h. We further hypothesized that the KIM-1 LFA results would be linear over at least five two-fold dilutions.

## Results

### Effect of storage time and temperature

**Fig. 1:**
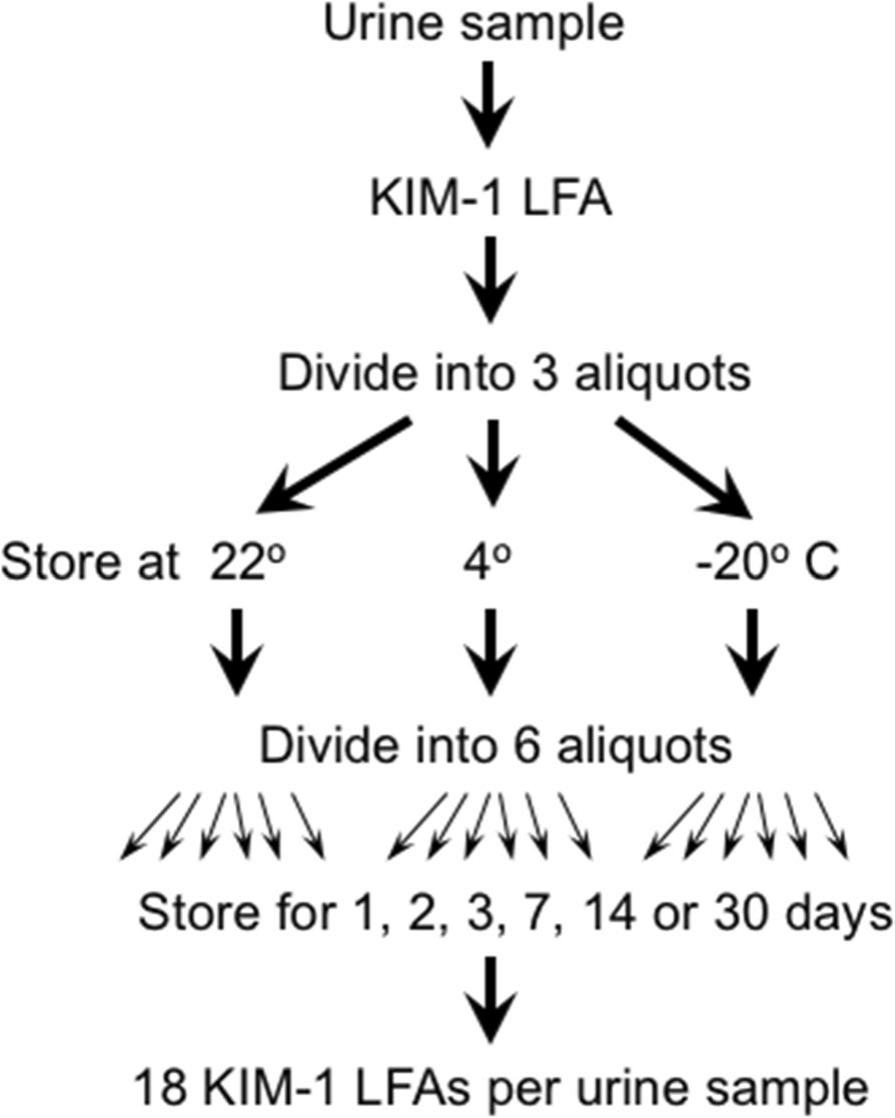
Schematic of study design for KIM-1 stability. Ten urine samples were used. Samples were stored at different temperatures and for different durations, as indicated

Eighteen aliquots of urine samples from 10 cats (Table S1) were stored for up to 30 days at different temperatures before performing the LFA (Fig. 1).

**Fig. 2:**
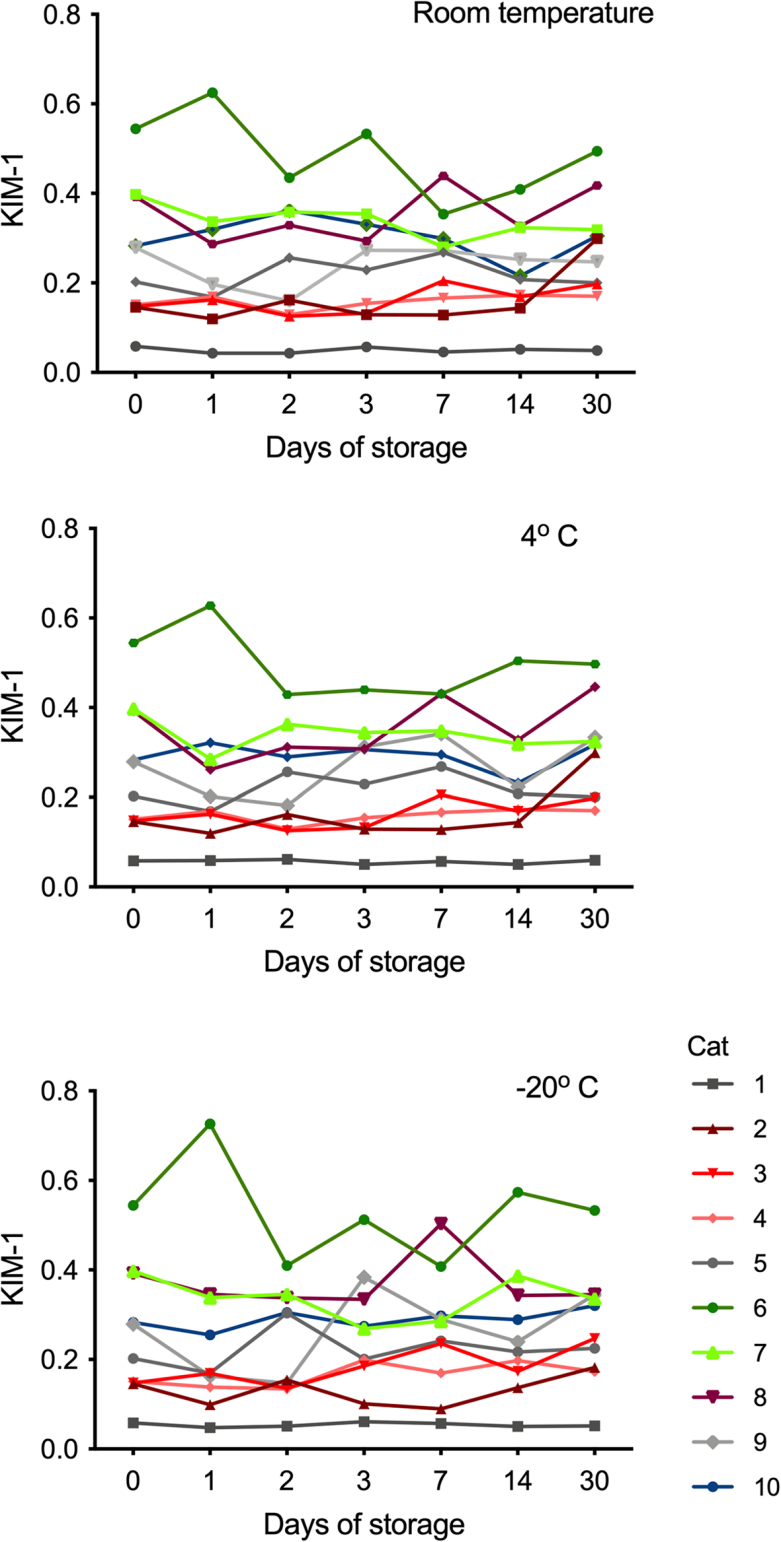
Effect of storage at different temperatures on detection of KIM-1 in feline urine. Different colored lines indicate individual urine samples. Day 0 is the day of urine collection. None of the values differed significantly from the results on Day 0, except the result on Day 30 at room temperature in urine from cat 2

Results obtained at different timepoints did not differ significantly to those from day 0 (Fig. 2). The only statistically significant difference (*p* = 0.045) was in a urine sample with relatively low KIM-1 that had been stored for 30 days at room temperature (cat 2, Fig. 2).

Upper and lower 95% tolerance and confidence intervals were calculated to assess the likelihood of differences in KIM-1 values to fall within the specified intervals over time relative to the day 0 value. The tolerance limits were narrower than the confidence intervals at each storage condition (Table S2).

### Intra-assay repeatability

**Fig. 3:**
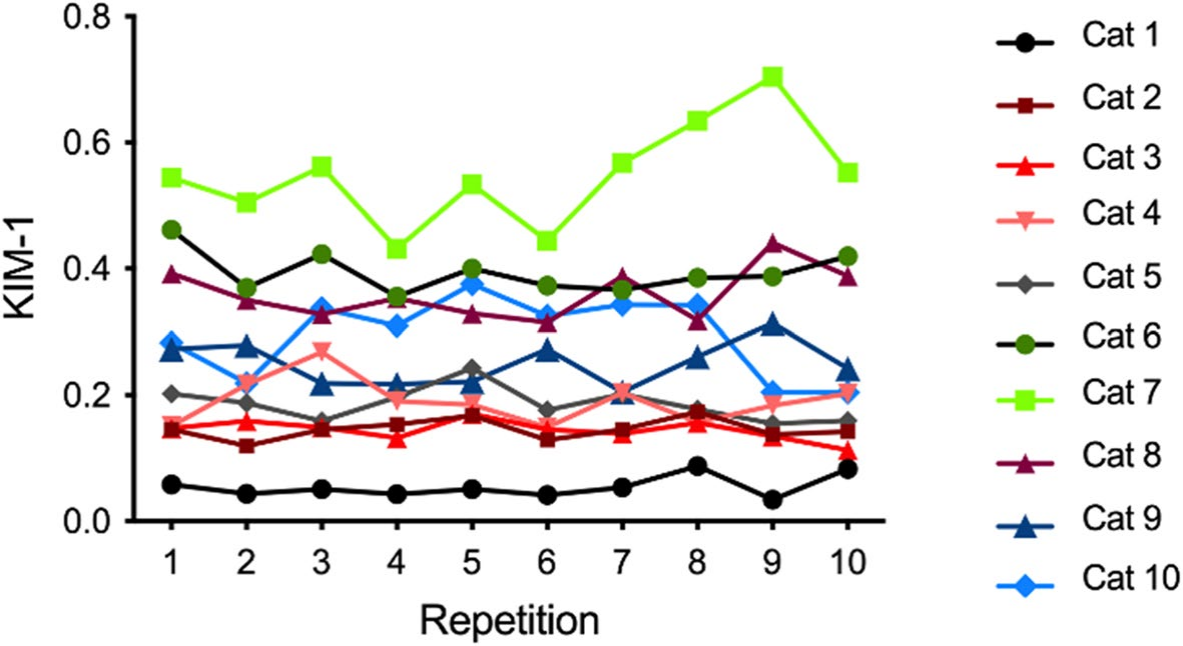
Intra-assay repeatability of measuring urine KIM-1 ten times in ten different urine samples. Results within samples did not differ significantly from each other

Results of KIM-1 measured repeatedly 10 times in 10 different urine samples (intra-assay variability) are shown in Fig. 3.

Intra-assay repeatability was evaluated by calculating the coefficient of variation (CV) for each sample, and the intra-assay correlation coefficient (ICC). The mean CV was 15.72% (range 8.22–32.13%), and the ICC was 0.92 (Table 1).

**Table 1:** Baseline urine KIM-1 values in cats measured with a lateral flow assay

Cat no	Mean KIM-1 ± SD^a,b^	CV^c^ (%)
1	0.055 ± 0.018	32.13
2	0.146 ± 0.016	10.99
3	0.145 ± 0.016	11.07
4	0.191 ± 0.036	18.77
5	0.186 ± 0.011	14.41
6	0.394 ± 0.032	8.22
7	0.548 ± 0.081	14.78
8	0.360 ± 0.041	11.37
9	0.250 ± 0.035	13.94
10	0.294 ± 0.063	21.56

^a^Each sample was analyzed consecutively 10 times

^b^Standard deviation

^c^Coefficient of variation

### Linearity

**Fig. 4:**
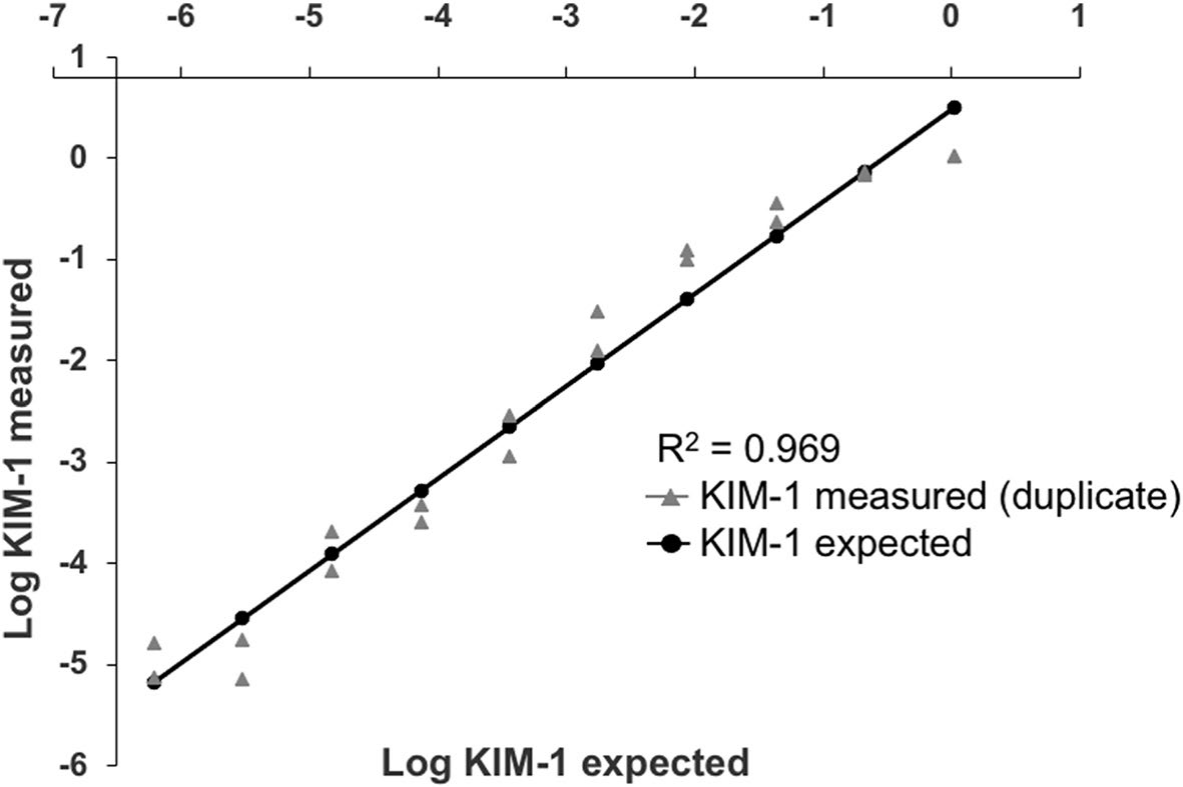
Measurement of KIM-1 in serially diluted urine. One urine sample (cat 11) with relatively high KIM-1 was diluted nine times two-fold, and then assessed in duplicate with the KIM-1 LFA. The results were plotted as the log of the measured values versus the predicted values. The solid line indicates the expected values; and the grey symbols reflect actual measurements

One urine sample with relatively high KIM-1 concentration was diluted two-fold from 1:2 to 1:512, and then analyzed. The calculated correlation coefficient (R^2^) for the measured versus the predicted results was 0.969 with a slope of 0.908 (Fig. 4).

The assay was linear over all dilutions, yielding the following equation for predicting concentration in diluted samples with a confidence interval of 0.9333 to 1.215 around the correlation coefficient:

$$0.908 \times \left(\mathrm{log KIM}-1\mathrm{ expected}\right) +0.4745$$

## Discussion

This study showed that storage of urine for at least 14 days at room temperature, 4^0^C or -20^0^C did not significantly affect detection of feline KIM-1 with this LFA. The assay yielded reliable and repeatable results that were linear until a 1:512 dilution. These characteristics suggest that the LFA is a robust test suitable for patient-side and retrospective analysis.

The first aim of this study was to determine the effect of up to 30 days of storage at different temperatures on the detectability of KIM-1 in urine. Urine samples were stored for up to 30 days, which was considered a suitable timeframe for clinical purposes. Effects of longer storage, as may be utilized for research purposes, remain to be established. Measurement of KIM-1 in the LFA depends on the interaction of a monoclonal antibody with a small region of the KIM-1 protein [22]. Since KIM-1 does not have to be intact to be recognized by such antibodies, it is likely that even partially degraded protein is still measured. This characteristic may account in part for reliable measurement up to 30 days. Consistent detection in stored urine distinguishes this LFA from other assays for detection of renal biomarkers, including KIM-1 in human samples [24–28]. Inconsistent biomarker detection over time has been attributed to interference by urine solutes, inconsistent sample centrifugation prior to storage, and the use of protease inhibitors [24–27]. In this study, urine samples were not centrifuged nor were protease inhibitors added prior to analysis or storage. While detection of KIM-1 over time was remarkably stable, there was some variability in values, in particular in the samples with higher KIM-1 on day 0. Reasons for this variability remain to be determined, but it may be possible that those urine samples with relatively high KIM-1 were in less optimal equilibrium with the capture antibody on the membrane, or that there was interference from other urine constituents present also at higher concentration, such as plasma proteins leaked across glomeruli, inflammatory mediators, or cell debris [29]. Future studies should aim to test matrix effects from urines containing a variety of potential interferents. Reasons for a statistically different value in one urine sample stored at room temperature for 30 days relative to baseline are underdetermined. Interference from accumulating bacteria or degenerating urine components is a potential cause. Hence, if urine is stored for longer than 14 days, refrigeration or freezing may be preferable to room temperature.

The methods for reporting the amount of urine constituents such as KIM-1 are highly variable. Some results are reported relative to urine creatinine concentration, while other results are reported as absolute values. With the LFA used in this study, KIM-1 values are reported as the ratio of the test value to a standardized positive control. While there are advantages and disadvantages of absolute versus relative quantification, normalization relative to urine creatinine concentration assumes that creatinine excretion is constant over time and consistent between individuals, and that there is a linear relationship between the biomarker and creatinine excretion [30–32]. Therefore, in theory normalization would account for urine dilution since it assumed that the biomarker and creatinine are equally and consistently changed in urine. While this approach was used historically, creatinine excretion has shown to decrease with progression of kidney disease, is variable over the course of 24 h, and is dependent on body mass [33]. Creatinine normalization, however, is still utilized in many studies to account, at least in part, for the influence of urine concentration. Glomerular filtration rate is also reduced and variable in AKI, which will decrease creatinine excretion and may therefore falsely increase the apparent concentration of a particular biomarker [30]. Quantification of a disease biomarker should be accurate, precise and reflective of the disease process, since the result often forms the basis for clinical intervention [22, 32, 34]. Therefore, it has been suggested that urinary biomarkers may be best expressed as absolute values during acute disease stages, and normalized to creatinine during chronic disease stages [31]. However, this approach also has limitations since patients with CKD may have ongoing subclinical acute injuries that affect the rate of urinary creatinine excretion. Another factor that may influence whether the concentration of a particular urine biomarker accurately reflects the extent and severity of tubular injury is urine flow. Severe AKI may result in widespread tubular cell death, occlusion of lumens by cell debris, and reduced urine volume [21]. Hence, reporting of both absolute and normalized values may be preferred until biomarker correlation with extent of kidney injury is better defined [32].

The second aim of this study was to characterize in vitro aspects of test performance. Intra-assay repeatability was high over a range of KIM-1 concentrations and as performed by a clinician simulating a patient-side scenario. Expected precision for diagnostic tests is typically considered excellent with a CV < 10%, good if 10-20%, accurate if 20-30% and unacceptable if > 30% [35]. The mean CV was 15.7%, and therefore consistent with ‘good’, although one urine sample had a CV > 30% and one other sample had a CV < 10%. Potential outliers were not removed, which may have increased the CV in the urine sample from cat 1.

Immunoassays typically have higher CVs [36] than spectrophotometric assays, and most POC tests yield only a yes/no result. The LFA technology presented here with RFID may have higher sensitivity and specificity than solely visual assessment and yields a quantitative result from a single rapid patient-side measurement. In this context, CVs < 20% are considered good but further investigation of repeatability across clinical applications and comparison to other methods is warranted [37].

The third aim of this study was to determine the linear range of the assay, which is important for clinical utility and may be helpful for establishing reference intervals that reflect renal health. A urine sample with relatively high KIM-1 (cat 11) was chosen to test nine twofold dilutions. Results showed a high degree of linearity over the entire range, suggesting that relatively small increments of different KIM-1 concentrations are reliably detected. To further define the upper limit of linear detection, urine with higher KIM-1 content may have to be tested. The upper limit of KIM-1 concentrations encountered in clinical settings remains to be determined. It was interesting to note that the sample from cat 11, a young and healthy cat anesthetized three times over two weeks, had the highest KIM-1 value. The cat had been anesthetized once for about 60 min to have a jugular catheter placed, then twice two and fourteen days later, respectively, for 180 min each time without surgical interventions, which may nevertheless have resulted in subclinical kidney injury.

There are limitations to be considered regarding the results from this study. Additional test characteristics of the KIM-1 LFA that should be determined are the effect of storage for longer than 30 days, inter-assay repeatability and interference. The total allowable error, reflecting the degree of change in an analyte that needs to be detected to enable clinical decision making, should be determined, in conjunction with evaluation of cats with and without AKI, as diagnosed using currently available modalities. KIM-1 values in a reference population of healthy cats are not yet determined, which limits interpretation regarding the magnitude of change expected for KIM-1 in different disease conditions. Similarly, available tests to detect AKI in cats are of limited sensitivity or practicality, which impacts equivalence testing. Measurement of GFR in relation to serial KIM-1 values may be the most suitable test for equivalence assessment.

In summary, while this LFA for feline KIM-1 has favorable in vitro characteristics and had value in research settings, it is important to note that the test may eventually augment but should not replace use of SCC and serum symmetric dimethylarginine concentration, or assessment of urine specific gravity, production, and sediment analysis. Rather, KIM-1 measurement should be considered as a new biomarker to be validated in clinical settings that may enhance the value of established diagnostic tests for detection of AKI.

## Conclusions

The feline urine KIM-1 LFA reliably detected KIM-1 in urine stored for ≥ 14 days at different temperatures. Studies to more closely define an interval of values reflective of renal health, and values associated with different disease conditions of the kidney, remain to be performed, as well as more complete validation prior to clinical implementation.

## Materials and methods

### Samples

Urine samples remaining from cats after routine urine collection by cystocentesis or catheterization on admission to the hospital, or collected from litter boxes during hospitalization, were screened with the feline KIM-1 LFA. Samples were submitted for urinalysis at the discretion of attending clinicians. In order to be included in this study, a volume of at least 8 mL of urine was required, the sample had to be collected < 6 h before analysis and stored refrigerated. Ten samples encompassing a range of KIM-1 values were chosen for analysis. Two samples originated from cats with a subcutaneous ureteral bypass (SUB) implanted to relieve ureteral obstruction, and one sample each from cats with hypertrophic cardiomyopathy, vertebral angiomatosis, portosystemic shunt and seizures, acute lymphocytic leukemia, suspected transient idiopathic cystitis, myelodysplastic syndrome, anemia and gastric foreign body (removed endoscopically). One additional sample with a high KIM-1 value (cat 11) was used for the dilutional study. This cat had been anesthetized once for 60 min and twice for 180 min over the course of 12 days as part of an unrelated research study (Supplementary Table 1). For that research, cats were premedicated with intramuscular hydromorphone (0.05 mg/kg) and midazolam (0.3 mg/kg), followed by intravenous propofol to effect for intubation. Anesthesia was maintained with isoflurane inhalant at 1.3 × minimal alveolar concentration for 180 min during which either 5 mL/kg of 6% hydroxyethyl plasma volume extender (Voluven, Fresenius Kabi, Toronto, ON) or 20 mL/kg of a balanced electrolyte solution (Plasmalyte, Baxter, Mississauga, ON) was administered intravenously. The urine sample was collected via a urinary catheter at the end of the 2^nd^ period of anesthesia.

### Sample analysis

All samples were analyzed by the same person in the same room in order to minimize operator and environmental variability. The LFA contains a membrane impregnated with a positive control and a site for application of the urine test sample in the test window of a small (~ 2 × 5 cm) disposable cassette. The assay cassette was placed on a flat surface, and 20 μL of urine was mixed with 400 μL of buffer in a 1.5 mL tube. The tube was inverted twice, and 150 μL of the mixed solution was applied to the membrane. All volumes were transferred with automated pipettes calibrated within the previous 12 months.^2^ After 15 min of incubation at room temperature, membrane-bound KIM-1 was visualized as a red line, and both the test line and positive control were quantified with the RFID reader. The test and control results were recorded, and the ratio of test to control values was calculated for statistical analysis. Of note, previously results of this assay were reported as the ratio of control/test values [22]. However, this was changed to a test/control format to align with the use of other ratios in laboratory medicine, such as the international normalized ratio in hemostasis testing.

### Effect of storage and temperature

Ten urine samples, five each with relatively low or relatively high KIM-1, were first divided into 3 aliquots, and then each was further allocated into 6 aliquots (Fig. 1). Aliquots were stored at either room temperature (22^0^ C), refrigerated at 4 ^0^C or frozen at -20 ^0^C for 1, 2, 3, 7, 14, and 30 days. Room temperature was set and maintained with a central temperature control system, and all temperatures were verified daily by thermometer measurements. At each of these time points, urine samples were allowed to come to room temperature, KIM-1 was measured using the LFA, and results were compared to the value on day 0 (day of urine collection).

### Intra-assay repeatability

The same operator also analyzed each of the above urine samples on day 0 consecutively ten times with the feline KIM-1 LFA to determine intraassay variability.

### Linearity

To determine the test's linear range, a urine sample with relatively high KIM-1 (Cat 11) was serially diluted twofold with buffer. Results were recorded for the neat sample and each of the following dilutions: 1:2, 1:4, 1:8, 1:16, 1:32, 1:64, 1:128, 1:256 and 1:512.

### Statistical analysis

Analyses were performed with statistical software.^3^ To assess the effect of storage and temperature on detection of KIM-1 in feline urine, a general linear mixed model was fit to the urine samples held at 3 different temperatures and 7 timepoints. Fixed effects included in the model were temperature and time and their interaction, and the random effect of individual cat was also included. Data were checked for normality with a Shapiro–Wilk test and examination of the residuals. Post hoc Dunnett’s test to compare each time point back to baseline was applied. Significance was set at *p* < 0.05.

To determine the intra-assay repeatability, ANOVA was used to calculate variance components for the variation between replicate measurements. The intra-assay correlation coefficient (ICC) as a measure of repeatability was calculated from these variances. Repeatability was reported as mean ± SD and CV (%).

Upper and lower 95% tolerance intervals were calculated to indicate where a single bias value (mean difference compared to baseline) will exist with 95% confidence, and upper and lower 95% confidence intervals were calculated to indicate the interval in which the mean of the bias for the group falls with 95% confidence for each storage condition (Suppl. Table 2) [38].

For the dilution study, a regression model was fit that tested linear and quadratic terms of expected relative to measured KIM-1 values. The data were plotted as log (ln) on the y- and x-axis. Examination of residuals and results of the Shapiro–Wilk test indicated that the data were normally distributed. The quadratic term was not significant and was removed from the final model.

## Supplementary Information

**Additional file 1:** **Supplementary Table 1.** Clinical and laboratory features of study cats. Urine from first 10 cats was used in the storage and intra-assay variability study, while urine from cat 11 was used in dilution study. **Supplemental Table 2.** Tolerance and confidence intervals of KIM-1 measurements in urine samples stored for up to 30 days.

**Additional file 2. **

## Data Availability

All data generated or analysed during this study are included in this published article and its supplementary information files. The raw data from this study are provided in Supplemental File 2.

## Abbreviations

AKI: Acute kidney injury
ACKD: Acute on chronic kidney disease
CC: Correlation coefficient
CI: Confidence interval
CKD: Chronic kidney disease
CV: Coefficient of variation
GFR: Glomerular filtration rate
IL-18: Interleukin-18
KIM-1: Kidney injury molecule-1
LFA: Lateral flow assay
NGAL: Neutrophil gelatinase-associated lipocalin
SCC: Serum creatinine concentration

## Footnotes

1 Bioassay Works LLC 10,075 Tyler Ct, Ijamsville, MD 21,754, US (301)874–8888.

2 Thermo Fisher Scientific Inc 5250 Mainway Burlington, ON L7L 5Z1 Canada (905)332–2000.

3 AS/STAT® 9.4. Cary, NC: SAS Institute Inc.

## References

[1] Marino CL, Lascelles BDX, Vaden SL, Gruen ME, Marks SL (2014). Prevalence and classification of chronic kidney disease in cats randomly selected from four age groups and in cats recruited for degenerative joint disease studies. J Feline Med Surg.

[2] Conroy M, Brodbelt DC, O'Neill D, Chang YM, Elliott J (2019). Chronic kidney disease in cats attending primary care practice in the UK: a VetCompass(TM) study. Vet Rec.

[3] IRIS Kidney [http://www.iris-kidney.com]

[4] Lawson JS, Liu HH, Syme HM, Purcell R, Wheeler-Jones CPD, Elliott J (2018). The cat as a naturally occurring model of renal interstitial fibrosis: Characterisation of primary feline proximal tubular epithelial cells and comparative pro-fibrotic effects of TGF-β1. PLoS ONE.

[5] Chen H, Dunaevich A, Apfelbaum N, Kuzi S, Mazaki-Tovi M, Aroch I, Segev G (2020). Acute on chronic kidney disease in cats: Etiology, clinical and clinicopathologic findings, prognostic markers, and outcome. J Vet Intern Med.

[6] Makris K, Spanou L (2016). Acute Kidney Injury: Definition, Pathophysiology and Clinical Phenotypes. Clin Biochem Rev.

[7] Von Hendy-Willson VE, Pressler BM (2011). An overview of glomerular filtration rate testing in dogs and cats. Vet J.

[8] Kidney Disease (2013). Improving Global Outcomes (KDIGO) CKD Work Group KDIGO 2012 Clinical Practice Guideline for the Evaluation and Management of Chronic Kidney Disease. Kidney Int Suppl.

[9] Cortellini S, Pelligand L, Syme H, Chang YM, Adamantos S (2015). Neutrophil Gelatinase-Associated Lipocalin in Dogs With Sepsis Undergoing Emergency Laparotomy: A Prospective Case-Control Study. J Vet Intern Med.

[10] Wang IC, Hsu WL, Wu PH, Yin HY, Tsai HJ, Lee YJ (2017). Neutrophil Gelatinase-Associated Lipocalin in Cats with Naturally Occurring Chronic Kidney Disease. J Vet Intern Med.

[11] Jepson RE, Syme HM, Markwell P, Miyazaki M, Yamashita T, Elliott J (2010). Measurement of urinary cauxin in geriatric cats with variable plasma creatinine concentrations and proteinuria and evaluation of urine cauxin-to-creatinine concentration ratio as a predictor of developing azotemia. Am J Vet Res.

[12] Bland SK, Côté O, Clark ME, DeLay J, Bienzle D (2014). Characterization of kidney injury molecule-1 in cats. J Vet Intern Med.

[13] Ghys LF, Paepe D, Lefebvre HP, Reynolds BS, Croubels S, Meyer E, Delanghe JR, Daminet S (2016). Evaluation of Cystatin C for the Detection of Chronic Kidney Disease in Cats. J Vet Intern Med.

[14] Ichimura T, Asseldonk EJ, Humphreys BD, Gunaratnam L, Duffield JS, Bonventre JV (2008). Kidney injury molecule-1 is a phosphatidylserine receptor that confers a phagocytic phenotype on epithelial cells. J Clin Invest.

[15] Zhang Z, Humphreys BD, Bonventre JV (2007). Shedding of the urinary biomarker kidney injury molecule-1 (KIM-1) is regulated by MAP kinases and juxtamembrane region. J Am Soc Nephrol.

[16] Bailly V, Zhang Z, Meier W, Cate R, Sanicola M, Bonventre JV (2002). Shedding of kidney injury molecule-1, a putative adhesion protein involved in renal regeneration. J Biol Chem.

[17] Peng H, Mao Y, Fu X, Feng Z, Xu J (2015). Comparison of biomarkers in rat renal ischemia-reperfusion injury. Int J Clin Exp Med.

[18] Vaidya VS, Ozer JS, Dieterle F, Collings FB, Ramirez V, Troth S, Muniappa N, Thudium D, Gerhold D, Holder DJ (2010). Kidney injury molecule-1 outperforms traditional biomarkers of kidney injury in preclinical biomarker qualification studies. Nat Biotechnol.

[19] Pais GM, Avedissian SN, O'Donnell JN, Rhodes NJ, Lodise TP, Prozialeck WC, Lamar PC, Cluff C, Gulati A, Fitzgerald JC (2019). Comparative Performance of Urinary Biomarkers for Vancomycin-Induced Kidney Injury According to Timeline of Injury. Antimicrob Agents Chemother.

[20] Shao X, Tian L, Xu W, Zhang Z, Wang C, Qi C, Ni Z, Mou S (2014). Diagnostic value of urinary kidney injury molecule 1 for acute kidney injury: a meta-analysis. PLoS One.

[21] Bland SK, Schmiedt CW, Clark ME, DeLay J, Bienzle D (2017). Expression of Kidney Injury Molecule-1 in Healthy and Diseased Feline Kidney Tissue. Vet Pathol.

[22] Bland SK, Clark ME, Côté O, Bienzle D (2019). A specific immunoassay for detection of feline kidney injury molecule 1. J Feline Med Surg.

[23] Flatland B, Freeman KP, Vap LM, Harr KE (2013). ASVCP guidelines: quality assurance for point-of-care testing in veterinary medicine. Vet Clin Pathol.

[24] van de Vrie M, Deegens JK, van der Vlag J, Hilbrands LB (2014). Effect of long-term storage of urine samples on measurement of kidney injury molecule 1 (KIM-1) and neutrophil gelatinase-associated lipocalin (NGAL). Am J Kidney Dis.

[25] Nauta FL, Bakker SJ, Lambers Heerspink H, de Zeeuw D, van Oeveren W, Bilo H, de Jong PE, Gansevoort RT (2012). Effect of frozen storage on urinary concentration of kidney damage markers. Am J Kidney Dis.

[26] Pennemans V, Rigo JM, Penders J, Swennen Q (2011). Collection and storage requirements for urinary kidney injury molecule-1 (KIM-1) measurements in humans. Clin Chem Lab Med.

[27] Schuh MP, Nehus E, Ma Q, Haffner C, Bennett M, Krawczeski CD, Devarajan P (2016). Long-term Stability of Urinary Biomarkers of Acute Kidney Injury in Children. Am J Kidney Dis.

[28] Parikh CR, Butrymowicz I, Yu A, Chinchilli VM, Park M, Hsu CY, Reeves WB, Devarajan P, Kimmel PL, Siew ED (2014). Urine stability studies for novel biomarkers of acute kidney injury. Am J Kidney Dis.

[29] Westgard J. Use and interpretation of common statistical tests in method comparison studies. Clin Chem. 2008;54(3):612. 10.1373/clinchem.2007.094060 18310152

[30] Waikar SS, Sabbisetti VS, Bonventre JV (2010). Normalization of urinary biomarkers to creatinine during changes in glomerular filtration rate. Kidney Int.

[31] Goldstein SL (2010). Urinary kidney injury biomarkers and urine creatinine normalization: a false premise or not?. Kidney Int.

[32] Tang KW, Toh QC, Teo BW (2015). Normalisation of urinary biomarkers to creatinine for clinical practice and research–when and why. Singapore Med J.

[33] Greenblatt DJ, Ransil BJ, Harmatz JS, Smith TW, Duhme DW, Koch-Weser J (1976). Variability of 24-hour urinary creatinine excretion by normal subjects. J Clin Pharmacol.

[34] Ralib AM, Pickering JW, Shaw GM, Devarajan P, Edelstein CL, Bonventre JV, Endre ZH (2012). Test characteristics of urinary biomarkers depend on quantitation method in acute kidney injury. J Am Soc Nephrol.

[35] Cui ZC (1989). Allowable limit of error in clinical chemistry quality control. Clin Chem.

[36] Beck SC, Lock RJ (2015). Uncertainty of measurement: an immunology laboratory perspective. Ann Clin Biochem.

[37] Lei R, Huo R, Mohan C (2020). Current and emerging trends in point-of-care urinalysis tests. Expert Rev Mol Diagn.

[38] Flouri M, Zhai S, Mathew T, Bebu I (2017). Tolerance limits and tolerance intervals for ratios of normal random variables using a bootstrap calibration. Biom J.

