# Supplementary material for: Effect of up to 30-days of storage at different temperatures on detection of feline kidney injury molecule-1 in urine
Source: BMC Vet Res. 2022 Nov 4;18:389. doi: 10.1186/s12917-022-03489-w (PMC9635161; doi:10.1186/s12917-022-03489-w)
Supplement: Supplementary file 1 — Additional file 1: Supplementary Table 1. Clinical and laboratory features of study cats. Urine from first 10 cats was used in the storage and intra-assay variability study, while urine from cat 11 was used in dilution study. Supplemental Table 2. Tolerance and confidence intervals of KIM-1 measurements in urine samples stored for up to 30 days. [file 12917_2022_3489_MOESM1_ESM.docx]

**Supplementary Table 1.** Clinical and laboratory features of study cats. Urine from first 10 cats was used in the storage and intra-assay variability study, while urine from cat 11 was used in dilution study.

| **Cat** | **Age (yr)** | **Breed^a^** | **Sex^b^** | **Condition** | **SCC^c^** | **USG^d^** | **Abnormal serum chemistry or CBC values** | **Abnormal urinalysis findings** | **KIM-1** |
| --- | --- | --- | --- | --- | --- | --- | --- | --- | --- |
| 1 | 10 | DSH | MN | Bilateral ureteral obstruction with CKD, sample collected at stable time-point post sub-ureteral bypass | 153 | 1.020 | Urea^e^ 14.9  Calcium^f^ 2.93 | Protein 2+  Blood 3+ | 0.058 |
| 2 | 10 | DLH | MN | End-stage obstructive hypertrophic cardiomyopathy | 95 | ND^g^ | None | ND | 0.146 |
| 3 | 2 | DSH | MN | Vertebral angiomatosis | 60 | ND | None | ND | 0.148 |
| 4 | 4 | Exotic SH | MN | Portosystemic shunt, seizures | ND | ND | Bile acids^h^ 18, 137  Urea 2.8  ALT^i^ 288 | ND | 0.151 |
| 5 | 16 | DSH | FN | Left ureteral obstruction with CKD, sample collected at stable time-point post sub-ureteral bypass | 195 | 1.024 | Urea 15.6  Calcium 3.06 | Protein 3+  Ketones 1+  Bilirubin 3+  *E. coli* 3+ | 0.202 |
| 6 | 6 | DSH | FN | Acute lymphocytic leukemia | 127 | ND | HCT^j^ 8  Urea 16.3  WBC^k^ 41.3 | ND | 0.545 |
| 7 | 2 | DSH | M | Colony blood donor cat with transient idiopathic cystitis | 103 | 1.052 | Glucose^l^ 13 | Trace protein  Bacteria 3+ | 0.397 |
| 8 | 2 | DSH | MN | Immune mediated anemia | 86 | ND | HCT 20% | ND | 0.392 |
| 9 | 13 | DLH | FN | Myelodysplastic syndrome, pleural effusion, chronic kidney disease | 205 | 1.014 | HCT 9%  Platelets 67^m^  Urea 14.5  ALT 134 | Protein 2+  Blood 3+  Bilirubin 2+  Granular casts | 0.279 |
| 10 | 2 | Bengal | MN | Gastric foreign body | 118 | ND | None | ND | 0.283 |
| 11 | 1 | DSH | M | Post general anesthesia | 137 | 1.041 | ND | Trace protein | 1.021 |

^a^ DSH, domestic short hair; DLH, domestic long hair

^b^ MN, male neutered; FN, female neutered

^c^ Serum creatinine concentration, reference interval (RI) 50 -190 μmol/L

^d^ Urine specific gravity

^e^ RI 6.0 – 12.0 mmol/L

^f^ RI 2.22 - 2.78 mmol/L

^g^ Not determined

^h^ RI pre 0 - 3, post 0 - 7 μmol/L

^i^ Alanine aminotransferase, RI 31 – 105 IU/L

^j^ Hematocrit, RI 28 - 49 L/L

^k^ White blood cell count, RI 4.2 - 13.0 x10^9^/L

^l^ RI 4.4 - 7.7 mmol/L

^m^ RI 93 - 514 x10^9^/L

**Supplemental Table 2.** Tolerance and confidence intervals of KIM-1 measurements in urine samples stored for up to 30 days.

| **Storage condition** | **Average bias^a^** | **Average observed error^b^** | **Lower 95% TI^c^** | **Upper 95% TI** | **Lower 95% CI^d^** | **Upper 95% CI** |
| --- | --- | --- | --- | --- | --- | --- |
| RT^e^ | 0.013 | 0.048 | -0.233 | 0.264 | -0.05 | 0.075 |
| 4 ^o^C | 0.026 | 0.044 | -0.216 | 0.241 | -0.052 | 0.066 |
| -20 ^o^C | 0.005 | 0.056 | -0.282 | 0.291 | -0.056 | 0.074 |

^a^ Mean difference between paired observations compared to baseline (day 0).

^b^ The calculated standard deviation of the bias

^c^ Tolerance interval in which a single bias value will exist with 95% confidence

^d^ Confidence interval in which the mean of the bias for the group falls with 95% confidence

^e^ Room temperature
